# Increased cell motility and invasion upon knockdown of lipolysis stimulated lipoprotein receptor (LSR) in SW780 bladder cancer cells

**DOI:** 10.1186/1755-8794-1-31

**Published:** 2008-07-22

**Authors:** Malene Herbsleb, Karin Birkenkamp-Demtroder, Thomas Thykjaer, Carsten Wiuf, Anne-Mette K Hein, Torben F Ørntoft, Lars Dyrskjøt

**Affiliations:** 1 Molecular Diagnostic Laboratory, Department of Clinical Biochemistry, Aarhus University Hospital, Skejby, Brendstrupgaardsvej 100, DK-8200 Aarhus N, Denmark; 2 BiRC – Bioinformatics Research Center, University of Aarhus, Hoegh-Guldbergs Gade 10, Building 1090, DK-8000 Aarhus C, Denmark

## Abstract

**Background:**

Mechanisms underlying the malignant development in bladder cancer are still not well understood. Lipolysis stimulated lipoprotein receptor (LSR) has previously been found to be upregulated by P53. Furthermore, we have previously found *LSR *to be differentially expressed in bladder cancer. Here we investigated the role of LSR in bladder cancer.

**Methods:**

A time course siRNA knock down experiment was performed to investigate the functional role of LSR in SW780 bladder cancer cells. Since LSR was previously shown to be regulated by P53, siRNA against *TP53 *was included in the experimental setup. We used Affymetrix GeneChips for measuring gene expression changes and we used Ingenuity Pathway Analysis to investigate the relationship among differentially expressed genes upon siRNA knockdown.

**Results:**

By Ingenuity Pathway analysis of the microarray data from the different timepoints we identified six gene networks containing genes mainly related to the functional categories "cancer", "cell death", and "cellular movement". We determined that genes annotated to the functional category "cellular movement" including "invasion" and "cell motility" were highly significantly overrepresented. A matrigel assay showed that 24 h after transfection the invasion capacity was significantly increased 3-fold (p < 0.02) in LSR-siRNA transfected cells, and 2.7-fold (p < 0.02) in TP53-siRNA transfected cells compared to controls. After 48 h the motility capacity was significantly increased 3.5-fold (p < 0.004) in LSR-siRNA transfected cells, and 4.7-fold (p < 0.002) in TP53-siRNA transfected cells compared to controls.

**Conclusion:**

We conclude that LSR may impair bladder cancer cells from gaining invasive properties.

## Background

Completion of the human genome project [1,2] together with development of microarray techniques have made it possible to investigate global changes in expression patterns that occur during bladder cancer development. Gene expression profiles associated with disease stage [3-7], disease progression [4,7,8], recurrence pattern [4], survival [6,9], and treatment response [10] have been delineated by several groups. Other studies have focused on the impact of single genes on tumorigenesis like the oncogenes *HRAS*, *FGFR3*, *ERBB2*, *CCND1*, and *MDM2*, and the tumor suppressors *CDKN2A*, *PTEN*, *TSC1*, and *DBC1 *(recently reviewed in [11]). Thus, knowledge on molecular alterations of single parameters is available, but the complex network of molecular events leading to invasive bladder cancer still requires further attention.

In this study we have focused on the lipolysis stimulated lipoprotein receptor gene (*LSR*), which is differentially expressed in bladder cancer. LSR was identified in 1992 as a receptor distinct from the low density lipoprotein receptor (*LDLR*) and low density lipoprotein-related protein 1 (*LRP1*) [12,13]. LSR binds ApoB and ApoE containing lipoproteins, especially chylomicrons and VLDL [12-15]. Unsaturated, long-chained (>12C) free fatty acids like oleate activate the receptor by causing a conformational change that expose the binding site [12-14,16]. The apparent number of LSR binding sites expressed at the surface of hepatocytes correlates negatively with plasma triglyceride levels measures at the postprandial stage [14]. After binding to LSR, the ligands are internalised and proteolytic degraded in lysosomes [12,13]. LSR binding is inhibited by lactoferrin [12-14], receptor associated protein (RAP) [17], and apoC-III [18], which all are reported to have hyperlipidemic effects. Previously, in an effort to identify primary TP53 targets, LSR was found to be upregulated by TP53 [19]. In another study, LSR was downregulated more than 2 fold upon TGF-β1 stimulation in wild-type cells with an active Smad4-TGF-β signalling pathway [20].

Here we investigated the participation of LSR in SW780 bladder cancer cells motility and invasion. We used a siRNA knock down in vitro assay where LSR and TP53 were knocked down individually and in combination in SW780 bladder cancer cells. Time course microarray analyses were used to measure the global consequences of the knockdown. *In silico *prediction of LSR regulated motility and invasion was experimentally verified by a matrigel assay.

## Methods

### Antibody synthesis

Polyclonal rabbit anti-LSR antibodies were raised against the peptide

[C]VTSLHEDDWRSRPSR, aa 386–400, BC000015.2 6.6.2006) conjugated to the carrier protein KLH (Keyhole limpet hemocyanin) (Eurogentec, Belgium). CLUSTALW analysis matched the peptide sequence to all three isoforms I1 NP_057009.3 (630 aa), I2 NP_991403.1 (649 aa) and I3 NP_991404.1 (581 aa) as shown in Additional File 1.

### Cloning and overexpression

cDNA was synthesized from total RNA from stage Ta tumors (non muscle invasive tumors) using SuperscriptTM cDNA synthesis kit (Invitrogen). cDNA was PCR amplified using primers sense 5'-ATGCAACAGGACGGACTTGG and antisense 3'-AGTCGGGAAAGTTTAGTCGTCTGA. The LSR PCR product was cloned into pcDNA 3.1 bidirectional (Invitrogen). Transient transfection with pcDNA 3.1_LSR isoform 1 and pcDNA 3.1_LSR isoform 3 of COS7 cells was achieved using FuGene (Roche) following the manufacturer's instructions yielding fragments with a 48 aa N-terminal truncation.

### Cell extraction, SDS gels and Western blots

COS7 cells transfected with pcDNA 3.1_LSR isoform 1 or isoform 3 were harvested by scraping and lysed in lysis buffer (50 mM Tris.HCl pH 8.0, 150 mM NaCl, 1 mM DTT, 1% Triton X-100, protease inhibitor Roche complete, EDTA free). 10–20 μg total protein samples were run in 12% SDS gels (Invitrogen) and transferred to PVDF membranes. Membranes were blocked with 3% w/v non-fat powder milk in PBS. The primary antibody was rabbit polyclonal anti-LSR (1:6000) and the secondary antibody goat anti-rabbit HRP conjugated (1:5000), DakoCytomation. The immunoreactive bands were visualised using ECL plus (Amersham biosciences) and an UVP ChemiDoc-It, Imaging system, UVP Inc.

### Immunohistochemistry

Immunohistochemistry was performed using 4 μm formalin fixed paraffin embedded (FFPE) sections of histological specimens as described previously [21]. We used a 1:3500 dilution of the rabbit polyclonal anti human LSR-antibody. Staining was scored independently by two observers (L.D and M.H). Staining categories were defined as no, weak, moderate or strong staining. In case of disagreement, the samples were re-evaluated and a consensus scoring was achieved.

### Knock down assay

SW780 cells (ATCC, CRL-2169) were grown in D-MEM medium (Invitrogen, cat. no. 31885-023) supplemented with 10% FCS (Invitrogen, cat.no. 10270-106) and 1% penicillin/streptomycin (Invitrogen, cat. no. 15140-122) at 37°C in a humidified environment with 5% CO_2 _atmosphere. The SW780 cells were selected for this study because they originate from a grade 1 tumor and hence resemble early steps in carcinogenesis before the cells become invasive. Cells were tested negative for Mycoplasma infection by MycoSensor PCR Assay set (Stratagene, cat. no. 302108, La Jolla, CA.).

Cells were harvested with Trypsin (cat.no. 25300-062). A nucleofector (Amaxa, Cologne, Germany) was used for cell transfections using Amaxa's standard program A23 [22]. For each siRNA construct 0.8 × 10^6 ^cells resuspended in 100 μl solution R (standard solution) were transiently transfected with a total of 1.2 μg siRNA using either mock siRNA (Non-targeting siRNA, Dharmacon, cat. no. D-001210-01), TP53 siRNA (Dharmacon, cat. no. M-003329-00), first LSR siRNA (LSR1, UCA AAG GUC AGG UCA GCA UTT, DNA Technology), second LSR siRNA (LSR2, siRNA ID 116788, Ambion), or 0.6 μg TP53 siRNA plus 0.6 μg LSR1. Transfected cells were grown for 18 h (one sample per siRNA construct), 48 h (4 samples per construct) or 72 h (one sample split in two technical replicates for further analysis).

Cells were harvested by scraping the flasks with 1 ml lysis buffer and total RNA was extracted from the cells using GenElute Mammalian Total RNA Miniprep Kit (Sigma-Aldrich, St. Louis, MO, cat.no. RTN350) according to the manufacturer's instructions.

### Microarray analysis of cell lines

Double stranded cDNA synthesis, in vitro transcription and labelling of cRNA, hybridisation and scanning was performed using standard procedures, see reference [5]. A total of thirty-five HG-U133 Plus 2 GeneChips (Affymetrix) were used.

### Data analysis of cell line microarray data

The arrays were normalised and expression measures generated using the GCRMA method [23] implemented in the ArrayAssist software (Stratagene). GCRMA stands for GeneChip robust multi-array analysis – see reference [22,24]. The mean expression of TP53 and LSR was calculated for each transfection and time point using the probes for LSR (208190_s_at) and TP53 (201746_at). There are two probesets for TP53 on the HG-U133 Plus 2 GeneChip, however, the other probeset (211300_s_at) is not specific for TP53. Then, for each time point three filters were applied to identify differentially expressed genes. The first filter removed non-expressed transcripts, in the way that transcripts were kept if all replicate samples from at least one siRNA construct were at least 6 (log2 scale number). The second filter was applied for each knockdown experiment (TP53, LSR1, LSR2, LSR1+TP53). It included transcripts, where the mean expression of mock transfected cells *or *the mean expression of knock down transfected cells was at least 6. An expression above 6 was defined as being above the noise threshold. Using these filters for the three time points gave twelve different subsets of probes with approximately 16,000 transcripts. A third filter was applied in Ingenuity, where a cut-off was applied to define "Focus Genes" as genes with a mean log ratio (compared to mock) of ≥ |1|. We used Excel (Microsoft) for filtering the data.

### Bioinformatics using Ingenuity Pathway Analysis

Each probe was mapped to its corresponding gene object in the Ingenuity Pathways Knowledge Base (IPKB). "Focus Genes" were superimposed the global molecular network developed from information contained in the IPKB. "Local networks" preferentially enriched of these Focus Genes were then generated based on their connectivity.

The "Functional Analysis of a network" identified the biological functions and/or diseases that were most significant to the genes in the network. The network genes associated with biological functions and/or diseases in the IPKB were considered for the analysis. Fischer's exact test was used to calculate a p-value determining the probability that each biological function and/or disease assigned to that network is due to chance alone.

The "Global Functional Analysis" identified the biological functions and/or diseases that were most significant to the entire data set. Genes from the dataset that met the log ratio cutoff of ≥ |1| and were associated with biological functions and/or diseases in the IPKB were considered for the analysis.

### Microarray data from human tissue samples

We used an independent dataset previously generated using HG-U133A Affymetrix GeneChips [5] on 87 bladder carcinomas (55 Ta tumors, 3 T1 tumors, and 29 T2–4 tumors) and 9 samples of normal urothelium. Gene expression measures were generated and data normalized using the GCRMA method [23] implemented in the ArrayAssist software (Stratagene). Mean expressions and log ratios were calculated for tumor and normal samples. A cut-off of log ratio ≥ |1| and p-value ≤ 0.05 was used to define "focus genes" in the network analysis.

### Realtime PCR

We measured expression of LSR (Assay ID Hs00210880_m1), EMP3 (assay ID Hs00171319_m1), AGR2 (assay ID Hs00180702_m1), CTGF (assay ID Hs00170014_m1) and GAPDH (Assay ID Hs99999905_m1) using standard procedures (Applied Biosystems). All samples were amplified in triplicate in an ABI Prism 7000 Thermal Cycler according to instructions of the manufacturer. TP53 expression was measured using a standard SYBR Green assay.

### Cell invasion assay

BD BioCoat Matrigel Invasion Chambers (BD Biosciences, cat. no. 354480) were used. To measure motility and invasion an equal number of control inserts (motility) and matrigel inserts (invasion) were used. 0.8 × 10^6 ^cells were transfected with AMAXA program A23 with 1.2 μg siRNA in 100 μl AMAXA solution R (standard solution in the Amaxa system) according to the instructions of the manufacturer. 25,000 transfected cells in a total volume of 0.5 ml were seeded into the top well of the inserts in serum free medium. The lower well was filled with 750 μl medium with serum. The transfected cells were allowed to grow and invade for 24 h and 48 h. We chose the 24 h timepoint instead of the 18 h timepoint that was used for the microarray analysis in order allow an effect of siRNA knockdown on the protein level. Then the non-invading cells were scraped from the upper surface of the membrane and the cells on the lower side were stained with Hemacolor staining set (Merck, cat. no. 1.11674) and four images covering the membrane were immediately taken under microscope. Each siRNA construct was tested in triplicate in both control inserts and matrigel inserts and the mean number of invading cells was calculated.

### Cell viability assay

A MTT (3-(4,5-Dimethylthiazol-2-yl)-2,5-diphenyltetrazolium bromide) ELISA assay (Roche, cat. no. 1-465-007) was used to test the impact of LSR and TP53 knockdown on cell viability. 0.8 × 10^6 ^SW780 cells were transfected with AMAXA program A23 with 1.2 μg siRNA in 100 μl AMAXA solution R for each siRNA construct. Cells were grown in quadruplicate in 96-wells microtiter plates with 12.000 cells per well in a total volume of 100 μl medium. After 24 h, 48 h or 72 h 10 μl MTT was added to a final conc. 0.5 mg/ml and cells were allowed to incubate for four hours. 100 μl solubilization solution was added to each well and the cells were lysed during incubation overnight. The absorbance was measured at 540 nm and 690 nm on a ELISA reader.

## Results

### Cloning and overexpression of LSR

**Figure 1:**
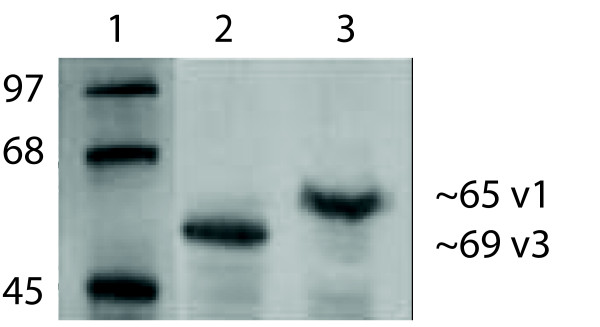
**Transient overexpression of truncated LSR isoforms 1 (v1) and 3 (v3) in COS7 cells.** Cell extracts were western blotted and identified by staining with polyclonal rabbit antibody. Lane 1; Mol. weight markers,; lane 2, LSR isoform 1; lane 3, LSR isoform 3.

From the data generated in a previous microarray screening of bladder tumors [5] we identified LSR as an interesting candidate gene for further analysis. Therefore, polyclonal rabbit anti-LSR antibodies were raised to characterise LSR protein expression. PCR amplification identified two transcript isoforms present in human bladder cancer, variants 1 and 3, while variant 2 was not amplified. Incubation of COS7 extracts overexpressing N-terminal truncated proteins of variant 1 or variant 3 showed that the antibody identified two LSR protein variants of about 65 kDa and 59 kDa (Figure 1) with a high specificity.

### Expression of LSR protein in bladder tumors

**Figure 2:**
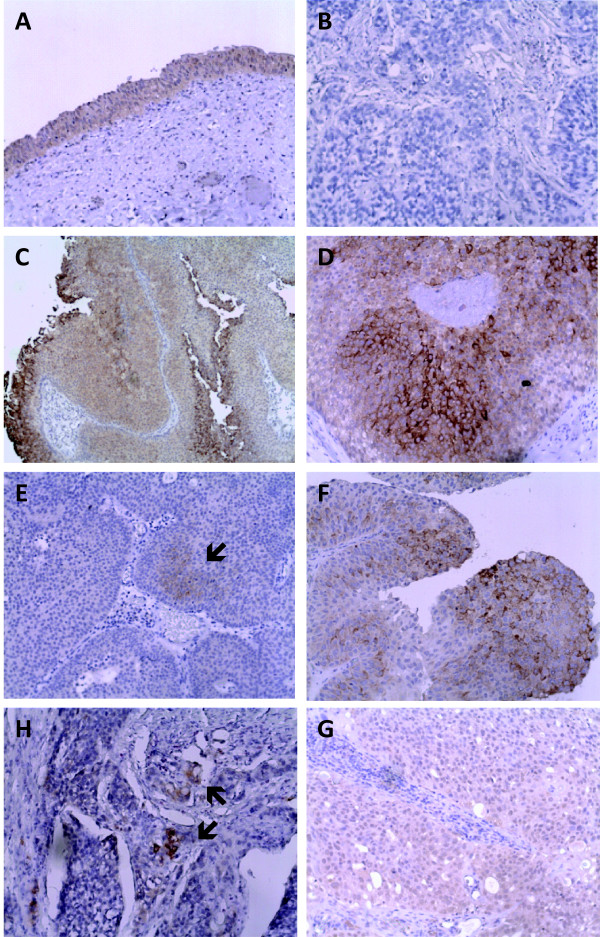
**Immunohistochemical analysis of LSR expression in normal urothelium and bladder cancer samples.** The protein was located in the cytoplasm in >50% of the cancer cells. Original magnification, ×20 (all except C), ×10 (C). A: Normal bladder urothelium (21859-99); medium LSR expression. B: Negative control stain without primary antibody of T3 grade 3 tumor (6268-96). C-D: Ta grade 2 bladder tumor (4232-97); median LSR expression. E: T1 grade 2 bladder tumor (6018-98); weak LSR expression. F: T1 grade 3 bladder tumor (5248-02); medium LSR expression. G: T3 grade 3 bladder tumor (6268-96); weak LSR expression. H: T2 grade 3 bladder tumor (1719-00); weak, overall LSR expression in cytoplasm and nucleus.

Immunohistochemical analysis of FFPE tissue sections from normal urothelium and bladder tumors showed a moderate to strong cytoplasmic expression of LSR in 100% of normal samples and 70% of Ta tumors (Figure 2). The expression was observed in nearly all urothelial cells. The overall number of cells staining positive for LSR was significantly decreasing in T1 and T2–4 tumors (Kendall's tau b, one-sided p-value = 0.0188), and only 38% of the muscle invasive samples showed moderate to strong cytoplasmic staining for LSR in the majority of cancer cells (Additional File 2). No staining was detected in connective tissue or lymphocytes.

### Microarray screening for LSR target genes

To investigate the function of LSR we used siRNA knockdown in the bladder cancer cell line SW780, which showed a high endogenous level of LSR expression. SW780 was transiently transfected with siRNA and two siRNAs against LSR were used (LSR1, LSR2) to evaluate eventual off-target effects [25]. A possible relationship between LSR and TP53 has been shown previously [19] and therefore siRNA against TP53 was also included. LSR was knocked down alone or in combination with TP53 (LSR1+TP53). All knock down experiments were compared to cells transfected with a non-targeting siRNA (mock).

**Figure 3:**
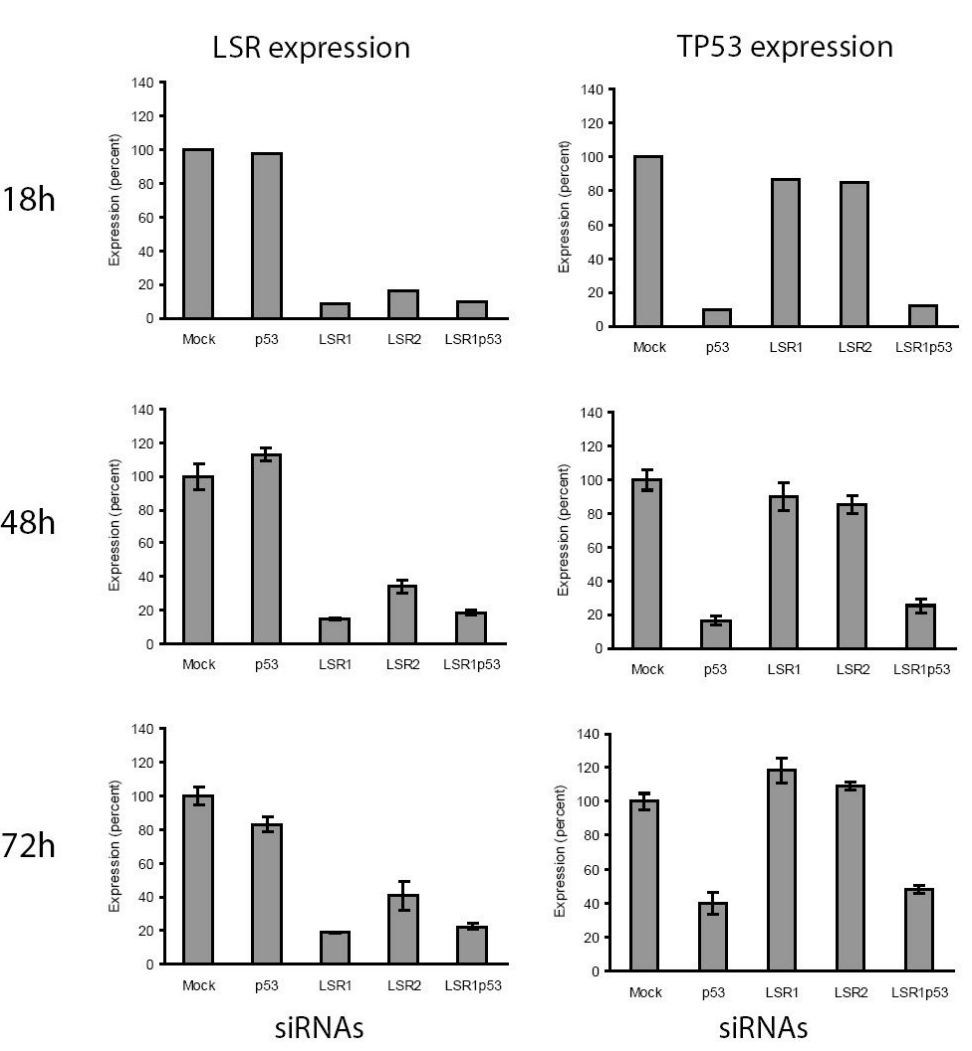
**Transient transfection of SW780 bladder cancer cells with a non-targeting siRNA (Mock), two different siRNAs against LSR (LSR1, LSR2), one siRNA against TP53 (TP53) and siRNA against LSR and TP53 in combination (LSR1+TP53).** Transfected cells were harvested 18 h (one sample per siRNA constructs), 48 h (4 samples per construct) or 72 h (one sample split in two technical replicates for further analysis) after transfection and gene expression was measured using microarray analysis (HG-U133 Plus 2 arrays, GeneChip, Affymetrix). The figure shows normalized mean expression of LSR (first graph column) and TP53 (second graph column). Error bars indicate +/- one standard deviation.

Cells were harvested after 18 h, 48 h and 72 h and microarray data documented that mean LSR expression was reduced significantly (Figure 3). Knockdown of TP53 transcripts had no impact on LSR expression at any time points. Measurements of TP53 expression showed that the siRNA against TP53 reduced the TP53 transcript level to 10% (18 h), 16% (48 h), and 40% (72 h) compared to mock transfected cells, while knockdown of LSR had no effect on TP53 expression.

### Overlapping effects of two LSR siRNAs

We found a large overlap in transcripts affected by LSR1 and LSR2, confirming that overall, the two LSR siRNAs had similar effect (Additional File 3). Furthermore, transcripts consistently affected over the three time points by each of the two siRNAs showed considerable overlap (Additional File 3); the sixteen common genes are shown in Table 1. The majority (44%) of these genes encoded proteins annotated to be located in the extracellular space like *CYR61*.

**Table 1:** Consistently differentially expressed genes at all measurements after LSR knock down by both LSR siRNAs (log2 scale).

Location		Gene		LSR1			LSR2	
			
			18 h	48 h	72 h	18 h	48 h	72 h
Cytoplasm	**AKAP12**	A kinase (PRKA) anchor protein (gravin) 12	1.2	1.8	1.9	1.1	2.1	2.1
								
Extracell. Space	**FST**	follistatin	1.8	3.2	2.8	1.9	3.9	3.6
	**APLP2**	amyloid beta (A4) precursor-like protein 2	1.3	1.6	1.1	1.3	1.6	1.2
	**IL1A**	interleukin 1, alpha	1.3	2.0	1.7	1.4	2.1	2.2
	**CYR61**	cysteine-rich, angiogenic inducer, 61	1.1	2.6	2.2	1.0	2.3	2.6
	**EDN1**	endothelin 1	1.2	3.1	1.8	1.2	2.7	2.6
	**GLIPR1**	GLI pathogenesis-related 1 (glioma)	1.4	2.2	2.6	1.7	2.8	2.8
	**PTHLH**	parathyroid hormone-like hormone	1.5	3.3	3.8	1.0	3.0	3.2
								
Nucleus	**EGR1**	early growth response 1	2.0	3.7	2.5	2.9	1.6	3.5
	**MYOCD**	myocardin	1.1	1.9	2.1	1.1	1.5	1.8
								
Plasma Membrane	**TSPAN8**	tetraspanin 8	-1.1	-1.9	-2.5	-1.9	-3.1	-3.0
	**MALL**	mal, T-cell differentiation protein-like	1.7	1.3	1.1	1.4	1.5	1.3
	**NEXN**	nexilin (F actin binding protein)	1.4	1.5	1.3	1.4	1.7	1.4
	**LSR**	lipolysis stimulated lipoprotein receptor	-3.6	-2.8	-2.4	-2.6	-1.6	-1.3
								
Unknown	TMCO1	transmembrane and coiled-coil domains 1	2.0	1.8	1.4	1.9	1.7	1.3
	TRIM31	tripartite motif-containing 31	-1.3	-2.0	-1.5	-1.2	-1.9	-1.7

Genes in bold are known by the Ingenuity Pathway Knowledge Base. Figures indicate log2 fold change at various time points.

### Knockdown effects and QPCR verification

A large number of genes were found to be differentially expressed upon knockdown (Additional File 4). 48 h after transfection, the microarray analysis showed a significant upregulation (1.6-fold, log2 scale) of the *EMP3 *expression (t-test, one-sided P = 9.9 × 10^-6^) and *CTGF *expression (2.6 fold)(t-test, one-sided P = 5.6 × 10^-5^), while *AGR2 *expression was significantly downregulated (-1.5 fold) (t-test, one-sided P = 1.3 × 10^-5^). QPCR analysis likewise showed a significant differential regulation of the expression of *EMP3 *(t-test, P = 7.0 × 10^-12^), *CTGF *(t-test, P < 1.0 × 10^-12^), and *AGR2 *(t-test, one-sided P = 8.4 × 10^-10^). This consistency was also found for the measurements 72 h after transfection.

### Bioinformatics analysis of the knockdown effects: Local network analysis

Using Ingenuity Pathway Analysis (IPA) we analysed the relationship between genes with a changed expression after knockdown of LSR, TP53 or both. "Local networks" preferentially enriched for "focus genes" were generated and ranked based on their connectivity (see Additional File 5).

**Figure 4:**
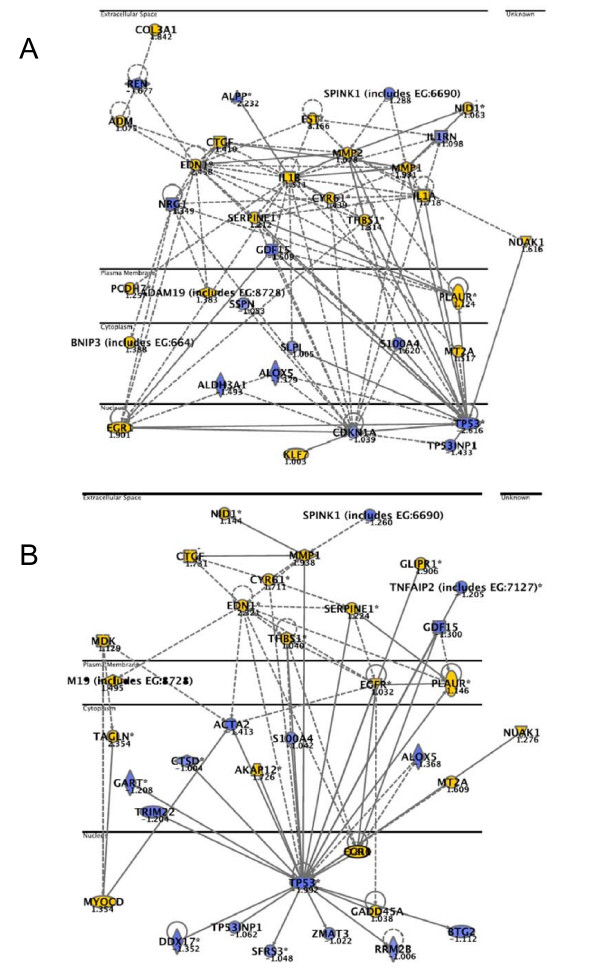
**Networks top-ranked by Ingenuity Pathway analysis after knockdown of TP53 (A) and LSR1+TP53 (B).** Coloured genes are upregulated (yellow) or downregulated (blue) more than 2-fold. "Focus genes" (bold) were defined as genes with a mean log ratio of ≥ |1| compared to mock transfected cells. The horizontal lines separate different cellular compartments.

Down-regulation of TP53: We found that the highest ranked local network upon downregulation of *TP53 *contained 35 focus genes (Figure 4A), which were annotated to the functional categories "cancer", "cellular growth and proliferation", and "cell death" (p-values between 6.6E-13 – 4.1E-12).

**Figure 5:**
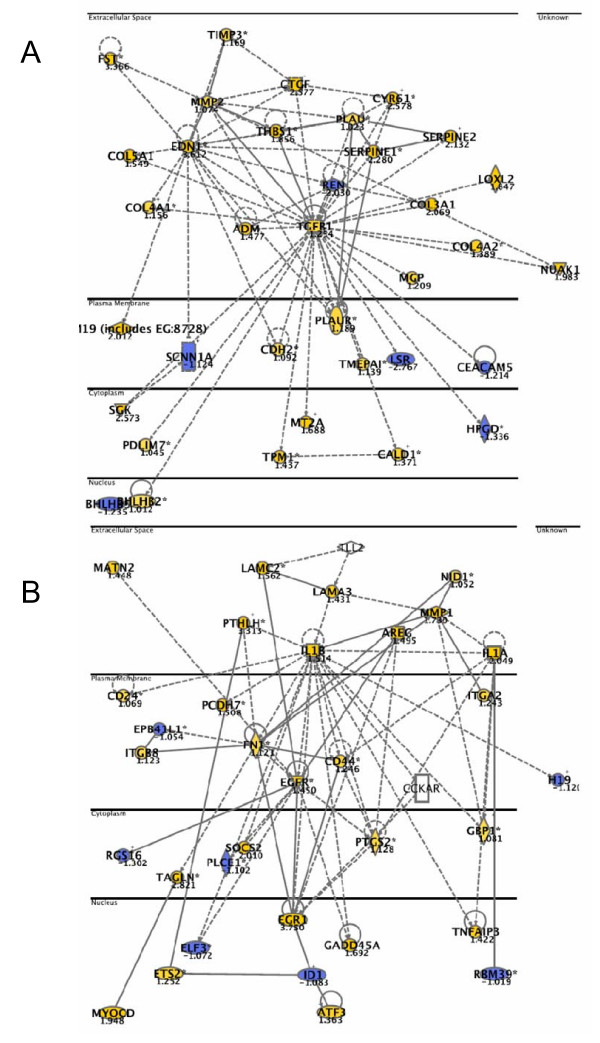
**Networks top-ranked by Ingenuity Pathway analysis after knockdown of LSR1 (A-B).** Coloured genes are upregulated (yellow) or downregulated (blue) more than 2-fold. "Focus genes" (bold) were defined as genes with a mean log ratio of ≥ |1| compared to mock transfected cells. The horizontal lines separate different cellular compartments.

**Figure 6:**
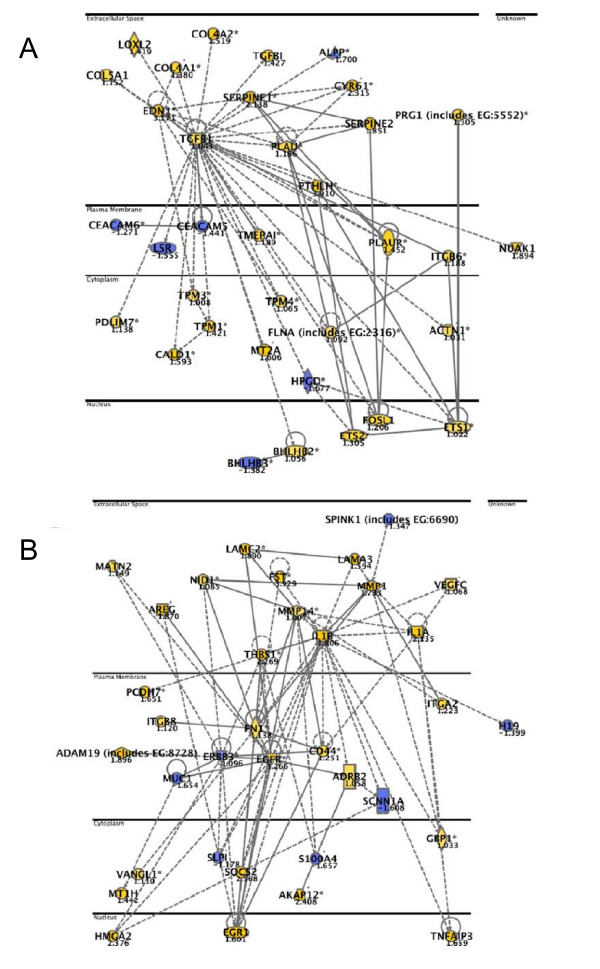
**Networks top-ranked by Ingenuity Pathway analysis after knockdown of LSR2 (A-B).** Coloured genes are upregulated (yellow) or downregulated (blue) more than 2-fold. "Focus genes" (bold) were defined as genes with a mean log ratio of ≥ |1| compared to mock transfected cells. The horizontal lines separate different cellular compartments.

Down-regulation of LSR: The highest ranked local network was annotated to functional categories like "cell growth and proliferation", "cell death", "cancer", and "cellular movement" dominated (p-values between 2.72E-14 – 1.8E-8). The majority of the genes in these networks were up-regulated (Figure 5, 6). *CYR61, EGR1*, and *AKAP12*, three of the genes constitutively regulated by both LSR siRNAs, were included in these networks (*CYR61*, Figure 5A and Figure 6A; *EGR1*, Figure 5B and Figure 6B; *AKAP12*, Figure 6B).

Down-regulation of TP53 and LSR in combination, 48 h: The highest ranked local network upon downregulation of TP53 and LSR contained 35 focus genes (Figure 4B). These genes were annotated to the functional categories "cancer", "cell death", and "cellular growth and proliferation" (p-values between 5.1E-11 – 6.0E-11).

### Bioinformatics analysis of the knockdown effects: Global functional analysis

Using IPA a "global functional analysis" identified the biological functions, processes or diseases that were most relevant for the entire dataset.

Knockdown of TP53: We investigated the global functional consequences of TP53 knockdown (Table 2). We found, that 48 h after transfection the functions affected were related to "cell-to-cell signalling" (P = 1.17 × 10^-10^), "organism survival" (P = 1.74 × 10^-9^), and "cellular growth and proliferation" (P = 1.29 × 10^-8^). The cell death/survival effect was even more prominent 18 h after transfection where all the three top-ranked functions were related to "cell death and survival" (P = 5.96 × 10^-9 ^– 3.72 × 10^-8^). This is consistent with the fact that TP53 expression was most prominent 18 h after transfection, where the highest knockdown effect was observed.

**Table 2:** "Global functional analysis" conducted using Ingenuity Pathway Analysis.

**Time after transfection**	**siRNA**	**Category**	**Process Annotation**	**Significance**
18 h	TP53	Cell Death	cell death of tumor cell lines	5.96E-09
		Organismal Survival	survival of mammalia	9.59E-09
		Cell Death	cell death of lung cancer cell lines	3.72E-08
		Organismal Survival	survival of mice	9.84E-08
		Cellular Growth and Proliferation	colony formation of eukaryotic cells	1.48E-07
	LSR1	Cancer	proliferation of tumor cell lines	3.78E-07
		DNA Replication, Recombination, and Repair	synthesis of DNA	4.61E-07
		Cell Death	apoptosis of cell lines	1.67E-06
		Cancer	cell death of tumor cell lines	1.95E-06
		Organismal Survival	survival of mammalia	2.29E-06
	LSR2	Cancer	growth of tumor cell lines	2.11E-07
		Cellular Growth and Proliferation	growth of cell lines	3.14E-07
		Cancer	proliferation of tumor cell lines	1.38E-06
		Cardiovascular System Development and Function	development of blood vessel	1.78E-06
		Cellular Growth and Proliferation	growth of eukaryotic cells	2.85E-06
	TP53+LSR1	Cellular Growth and Proliferation	colony formation of eukaryotic cells	5.07E-10
		Organismal Survival	survival of mammalia	8.79E-09
		Cellular Growth and Proliferation	colony formation of cell lines	1.11E-08
		Cell Death	cell death of cell lines	3.66E-08
		Cancer	colony formation of tumor cell lines	1.11E-07

48 h	TP53	Cell-To-Cell Signaling and Interaction	activation of eukaryotic cells	1.17E-10
		Organismal Survival	survival of rodents	1.74E-09
		Cellular Growth and Proliferation	proliferation of cell lines	1.29E-08
		Cellular Growth and Proliferation	growth of central nervous system cells	1.76E-08
		Cellular Growth and Proliferation	colony formation of cells	3.72E-08
	LSR1	Cellular Movement	cell movement	3.26E-12
		Cellular Movement	invasion of eukaryotic cells	2.31E-11
		Cellular Movement	invasion of cell lines	1.61E-09
		Cellular Movement	cell movement of cell lines	3.84E-09
		Cellular Movement	cell movement of endothelial cells	4.24E-09
	LSR2	Cellular Movement	cell movement	2.72E-15
		Cellular Growth and Proliferation	growth of tumor cell lines	4.45E-12
		Cellular Movement	cell movement of eukaryotic cells	4.54E-12
		Cell-To-Cell Signaling and Interaction	adhesion of cells	4.78E-12
		Cellular Movement	cell movement of cell lines	7.54E-12
	TP53+LSR1	Renal and Urological Disease	renal and urological disorder of rodents	3.87E-08
		Renal and Urological Disease	renal and urological disorder of rats	1.05E-07
		Cancer	growth of tumor cell lines	1.99E-07
		Cellular Growth and Proliferation	colony formation of eukaryotic cells	5.94E-07
		Cellular Growth and Proliferation	colony formation of cell lines	1.18E-06

72 h	TP53	Tissue Development	development of tissue	5.91E-08
		Tissue Development	angiogenesis of chorioallantoic membrane	8.76E-08
		Renal and Urological Disease	renal and urological disorder of rodents	1.01E-07
		Cancer	angiogenesis of tumor	1.82E-07
		Cellular Movement	cell movement	1.99E-07
	LSR1	Cellular Movement	cell movement	8.99E-11
		Cellular Movement	migration of eukaryotic cells	2.41E-09
		Cellular Movement	cell movement of eukaryotic cells	2.46E-09
		Cellular Movement	cell movement of cell lines	5.27E-09
		Cellular Movement	migration of tumor cell lines	1.72E-08
	LSR2	Cellular Movement	cell movement	5.60E-15
		Cellular Movement	cell movement of eukaryotic cells	2.46E-13
		Cellular Movement	migration of eukaryotic cells	7.57E-13
		Cellular Growth and Proliferation	growth of tumor cell lines	5.66E-12
		Tissue Development	formation of tissue	8.77E-12
	TP53+LSR1	Cellular Movement	migration of eukaryotic cells	4.19E-10
		Cellular Movement	cell movement	8.74E-10
		Cellular Movement	cell movement of eukaryotic cells	1.57E-09
		Cancer	angiogenesis of tumor	6.86E-09
		Renal and Urological Disease	renal and urological disorder of rodents	8.15E-09

The list includes the annotation of the differentially expressed genes upon knockdown. The likelihood that the association between a set of differentially expressed genes and a given function was due to chance was calculated using the right-tailed Fisher's exact test. The biological functions or processes annotated to affected genes are listed according to their significance.

Knockdown of LSR: We found that the three most significant functions or processes after knockdown with LSR1 were all related to "cellular movements" (cell movement, invasion of eukaryotic cells, and invasion of cell lines). This was highly significant with P-values between 3.26 × 10^-12 ^and 1.61 × 10^-9^. For knock down of LSR with LSR2 the three top ranked functions were related to "cell movement" (P = 2.72 × 10^-15^), "growth of tumor cell lines" (P = 4.45 × 10^-12^), and "cell movement of eukaryotic cells" (P = 4.54 × 10^-12^). The same tendency was found at 72 h where all top-three ranked functions affected by knockdown of LSR by either LSR siRNA were related to "cellular movement" (P = 5.60 × 10^-15 ^– 2.46 × 10^-9^). See Table 2.

Knockdown of LSR and TP53: For knock down of LSR and TP53 expression in combination the most significant function affected was related to "renal and urological disorder" (P = 3.87 × 10^-8^). This was even more significant 72 h after transfection (P = 8.15 × 10^-9^). Genes involved in that process were *EDN1*, *GADD45A*, *IL1A*, *MT2A*, *PTHLH*, *REN*, *RRM2B*, *SERPINE1*, *THBS1*, *TGFB1*, *UPK2*, and *VEGF*. Other highly significant functions were "cancer growth of tumor cell lines" (P = 1.99 × 10^-7^), and "cellular growth" and "proliferation" (colony formation of eukaryotic cells) (P = 5.94 × 10^-7^). See Table 2.

In conclusion, our *in vitro *experiment indicated that knock down of LSR affected genes known to be involved in cancer and especially cellular movements like invasion, migration and motility.

### Verification of the invasive potential of LSR knockdown

**Figure 7:**
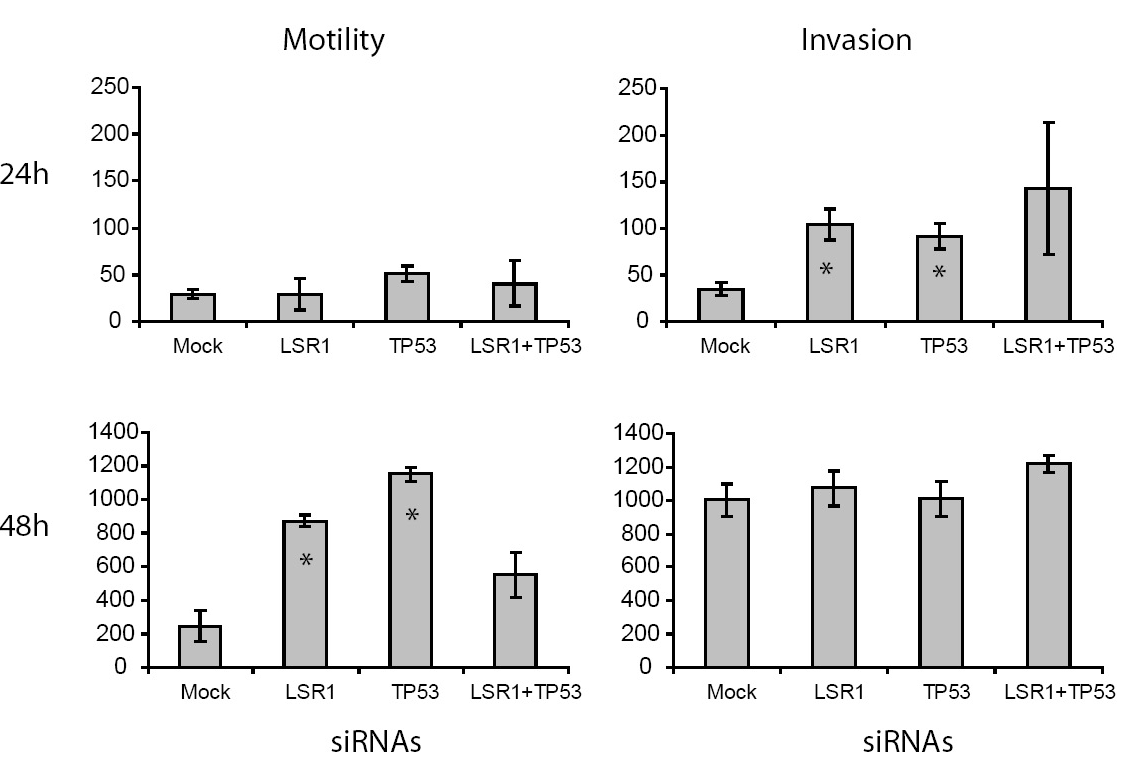
**Cell invasion assay.** Cells were transfected with siRNAs (Mock, LSR1, TP53, LSR1+TP53) and were allowed to grow for 24 h or 48 h in control inserts (three samples per transfection) or in matrigel matrix inserts (three samples per transfection). The mean number of cells passing the inserts was calculated. Error bars indicate +/- one standard error. Asterisks (*) indicate transfections where the number of cells passing the membrane was significantly different from the mock transfected cells.

Using a matrigel assay we measured the motility and invasion capacity after knockdown of LSR or TP53 to investigate the *in silico *predicted functional role of LSR. The SW780 bladder cancer cell line was transfected with siRNAs against LSR (LSR1), TP53 and LSR1+TP53. A mock siRNA was used as control. The experiment showed that 24 h after transfection, the invasion capacity was significantly elevated 3-fold in LSR1 knock-down cells (P = 0.0194) and 2.7-fold in TP53 knock-down cells (P = 0.0203) compared to mock transfected cells (Figure 7). After 48 h cell motility was elevated 3.5-fold in LSR1 knock down cells (P = 0.0044) and 4.7-fold in TP53 knock-down cells (P = 0.0020) compared to mock transfected cells (Figure 7). There was no significant effect on motility 24 h after transfection, and no effect on invasion 48 h after transfection. There was no significant effect of knockdown with siRNAs against LSR and TP53 in combination.

### Measurement of cell viability after knockdown of LSR and TP53

Since our "global functional analysis" showed that knockdown of TP53 transcripts influenced genes involved in cell death and cell survival we conducted a viability assay to measure if there were any difference in number of viable cells. Further, we wanted to see, if the invasion capacity of LSR knock down cells was due to difference in viability. We used an assay based on MTT, which is cleaved by viable cells. SW780 cells were transfected in quadruplicate with siRNAs against mock, LSR, TP53 and TP53+LSR. However, there was no significant viability difference between any of the knockdowns and the mock transfected cells (Additional File 6). Consequently, the invasive capacity upon LSR knockdown was not caused by a difference in number of viable cell.

### Cellular movement network using cell line and tumor derived expression data

**Figure 8:**
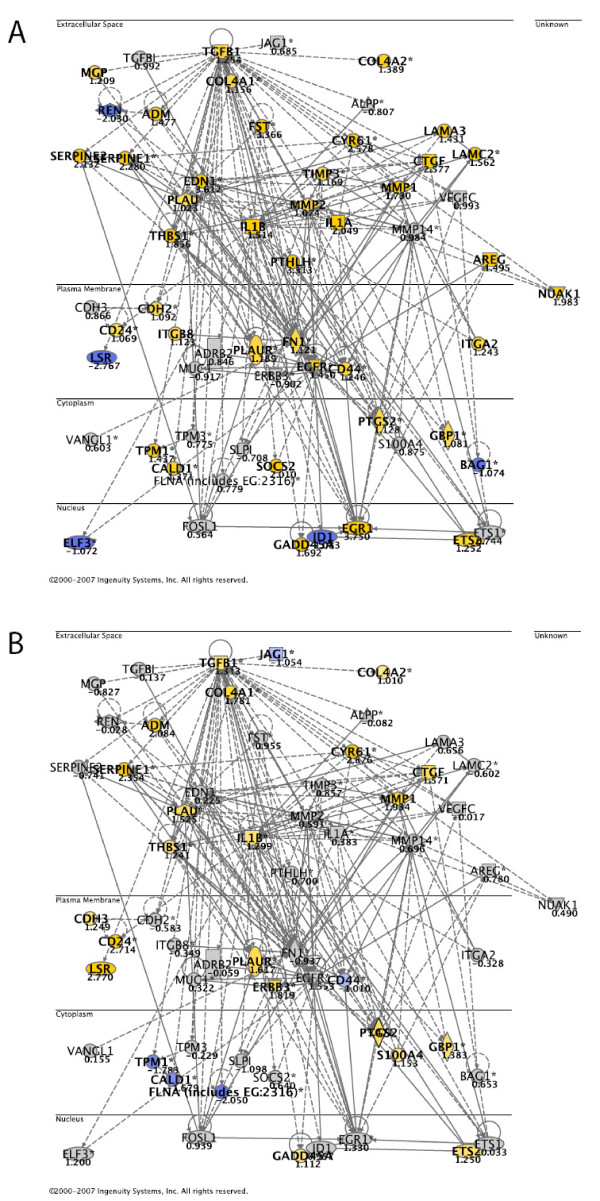
**Cellular movement and invasion network.** A; log ratios of mean expression 48 h after knockdown with LSR1 compared to mean expression 48 h after knockdown with mock siRNA were overlaid. B; microarray data from 87 bladder tumors and 9 normal urothelium samples superimposed the cell movement network. Yellow represents up-regulated genes and blue represents down-regulated genes. Genes not fulfilling the cut-off criteria are not colored.

Since elevated invasion potential seemed to be an important functional consequence of LSR knockdown, we constructed a "cellular movement" network. 64 genes, which were differentially expressed upon knockdown of LSR and which were significantly related to the gene categories cellular movements, invasion and migration with p-values below 1.0 × 10^-10 ^were selected for network generation. 60 of these could be directly or indirectly connected using IPA. Then log ratios of mean expression 48 h after knockdown with LSR1 compared to mean expression after knockdown with mock siRNA were superimposed (Figure 8A). Further, from the microarray data from the clinical specimens we calculated mean expression values and log ratios between tumor and normal urothelium samples, for the same gene set. These data were superimposed the same cell movement network (Figure 8B). The correlation between the gene expression for the two dataset was marginally significant (Kendall's tau b, one-sided p-value = 0.0526). However, the complexity of the clinical samples makes this an interesting finding.

## Discussion

We investigated the functional role of LSR in vitro by transient transfection of LSR and TP53 siRNAs into a bladder cancer cell line (SW780) followed by microarray measurements of gene expression. We used Ingenuity Pathway Analysis to investigate the relationship among differentially expressed genes upon knockdown and thereby, to generate hypotheses and to suggest directions for further phenotypic analyses. We identified six gene networks containing genes mainly related to the functional categories "cancer", "cell death", and "cellular movement". Further, by investigating the entire dataset, we determined that genes annotated to the functional category "cellular movement", including "invasion" and "cell motility", were highly significantly overrepresented among the differentially expressed genes after LSR knockdown. We investigated the role of these genes in a phenotypic matrigel assay, which showed that the invasion capacity was significantly elevated 24 h after transfection with LSR1 or TP53 siRNAs compared to mock transfected cells, while the motility capacity was significantly elevated 48 h after transfection with LSR1 or TP53. The motility and invasion capacity was controlled for cell viability. These results indicate that in the urinary bladder LSR plays a role in cancer development.

The role of LSR has been thoroughly investigated in humans, rats and mice [12-15,17,26]. LSR is mainly expressed in liver membranes [13,15,17] and is believed to mediate clearance of chylomicrons after activations of free fatty acids [13,14]. However, other functions may exist, since LSR is also expressed in other tissues [20,27-29]. LSR expression has been shown in tumor tissue like ovarian cancer, where LSR was one of thirty genes identified as a potential tumor marker [29].

A relationship between LSR, transforming growth-factor β1 (TGF-β1) and Smad4 has been investigated in relation to cell movements measured by a wound closure assay [20]. TGF-β induced signalling can be induced in a Smad4 dependent and independent way. Briefly, the experiment showed that in presence of Smad4, TGF-β1 stimulation depressed LSR expression and induced cell movements. Upon TGF-β1 stimulation in Smad4 knocked down cells, LSR was not reduced and migration was delayed. TGFB1 has also been associated with cell migration [30].

In our in vitro experiment we found that TGF-β1 was upregulated more than 2-fold after knockdown of LSR (Figure 8A). Similar, we saw that TGF-β1 expression was upregulated in vivo. Further, our invasion assay showed that the invasion and migration capacity was elevated when LSR was downregulated, consistent with the findings by Jazag *et al*. These results suggest that TGF-β1 expression can be regulated by the LSR level. Smad4 was not differentially expressed and was not included in the analysis.

The data document that it is quite useful to conduct time-course microarray studies and to use in-silico data mining, such as network and functional network analysis, to understand the effect of single molecules. In the present case we would not have identified the invasion and motility effect of LSR had it not been suggested by the pathway analysis.

In an effort to handle possible off-target effects we included two different siRNAs against LSR. Both siRNAs demonstrated a comparable knockdown effect of LSR. Sixteen genes were constitutively regulated over time by both LSR siRNAs. Among these were *CYR61*, which has been shown to be overexpressed and associated with advanced stages of breast cancer [31], and *EGR1*, which is believed to be a direct regulator of tumor suppressors like *TGFB1*, *TP53 *and *PTEN *[32]. *EGR1 *was upregulated upon knockdown of LSR *in vitro *and upregulated when comparing tumor samples with normal urothelium samples, though not significantly. Finally, the "local network analysis" and the "global functional analysis" conducted in IPA showed overall the same findings. Therefore, we believe that our LSR siRNAs mainly target the same LSR transcript and that possible off-target effects are minimal.

We included knockdown of TP53 for two reasons: First, LSR was previously identified as a potential primary target of TP53 [19]. In our experiment we could not see any effect on LSR transcription after TP53 knockdown and vice versa. Secondly, we included knockdown of TP53 as a control model in our *in silico *investigations. Both the "local network analysis" and the "global functional analysis" showed that genes affected by TP53 knockdown could be related to cancer and cell death.

## Conclusion

LSR may be involved in both invasion, and cellular movement in bladder cancer. Future research will document whether LSR-like molecules could be used therapeutically in bladder cancer treatment.

## Competing interests

The authors declare that they have no competing interests.

## Authors' contributions

MH, KB–D, TT, CW, TFØ and LD participated in the study design. MH performed most of the microarray analyses and invasion assays. KBD participated in the IHC work. MH drafted the paper. MH, KBD, TFØ and LD participated in the final preparation of the paper. All authors read and approved the final manuscript.

## Pre-publication history

The pre-publication history for this paper can be accessed here:



## Supplementary Material

Additional file 1 Antibody generation. CULSTALW1.83 analysis of the three isoforms and the peptide used for immunization of rabbits. Click here for file

Additional file 2 IHC results. Immunohistochemical staining of LSR in 40 bladder tumor samples. Click here for file

Additional file 3 Comparison of number of genes differentially expressed. A: Number of probes differentially expressed (LR ≥ |1|) by both LSR siRNAs (LSR1 and LSR2) at three different time points. B: Number of probes differentially expressed (LR ≥ |1|) by the same LSR siRNA (LSR1 or LSR2) at three different time points. C: Number of probes differentially expressed (LR ≥ |1|) by both LSR siRNAs (LSR1 and LSR2) at all three time points. Click here for file

Additional file 4 Gene expression data. Genes found to be differentially expressed upon knockdown. Click here for file

Additional file 5 Functional categories. Overview of functional categories related to differentially expressed genes in top ranked "local networks". Click here for file

Additional file 6 Viability assay. A MTT ELISA assay was used to test the impact of LSR and TP53 knockdown on cell viability. SW780 cells were transfected in quadruplicate with siRNAs against mock, LSR, TP53 and TP53+LSR. 24 h, 48 h or 72 h after transfection MTT was added and cells were allowed to incubate for four hours. Then the cells were lysed and the absorbance was measured at 540 nm and 690 nm (reference) on an ELISA reader. Click here for file
