# Supplementary material for: Increased cell motility and invasion upon knockdown of lipolysis stimulated lipoprotein receptor (LSR) in SW780 bladder cancer cells
Source: BMC Med Genomics. 2008 Jul 22;1:31. doi: 10.1186/1755-8794-1-31 (PMC2492871; doi:10.1186/1755-8794-1-31)
Supplement: Additional file 2 — IHC results. Immunohistochemical staining of LSR in 40 bladder tumor samples. [file 1755-8794-1-31-S2.doc]

**Additional file 2**
