# Supplementary material for: Increased cell motility and invasion upon knockdown of lipolysis stimulated lipoprotein receptor (LSR) in SW780 bladder cancer cells
Source: BMC Med Genomics. 2008 Jul 22;1:31. doi: 10.1186/1755-8794-1-31 (PMC2492871; doi:10.1186/1755-8794-1-31)
Supplement: Additional file 3 — Comparison of number of genes differentially expressed. A: Number of probes differentially expressed (LR ≥ |1|) by both LSR siRNAs (LSR1 and LSR2) at three different time points. B: Number of probes differentially expressed (LR ≥ |1|) by the same LSR siRNA (LSR1 or LSR2) at three different time points. C: Number of probes differentially expressed (LR ≥ |1|) by both LSR siRNAs (LSR1 and LSR2) at all three time points. [file 1755-8794-1-31-S3.doc]

**Additional file 3**

A

B

C
