# Supplementary material for: Increased cell motility and invasion upon knockdown of lipolysis stimulated lipoprotein receptor (LSR) in SW780 bladder cancer cells
Source: BMC Med Genomics. 2008 Jul 22;1:31. doi: 10.1186/1755-8794-1-31 (PMC2492871; doi:10.1186/1755-8794-1-31)
Supplement: Additional file 6 — Viability assay. A MTT ELISA assay was used to test the impact of LSR and TP53 knockdown on cell viability. SW780 cells were transfected in quadruplicate with siRNAs against mock, LSR, TP53 and TP53+LSR. 24 h, 48 h or 72 h after transfection MTT was added and cells were allowed to incubate for four hours. Then the cells were lysed and the absorbance was measured at 540 nm and 690 nm (reference) on an ELISA reader. [file 1755-8794-1-31-S6.doc]

**Additional file 6**
